# Identification of Novel Predictive Biomarkers for Endometrial Malignancies: *N*-Acylethanolamines

**DOI:** 10.3389/fonc.2019.00430

**Published:** 2019-06-11

**Authors:** Thangesweran Ayakannu, Anthony H. Taylor, Timothy H. Marczylo, Mauro Maccarrone, Justin C. Konje

**Affiliations:** ^1^Reproductive Sciences Section, Department of Cancer Studies and Molecular Medicine, University of Leicester, Leicester, United Kingdom; ^2^Gynaecology Oncology Cancer Centre, Liverpool Women's NHS Foundation Trust, Liverpool Women's Hospital, Liverpool, United Kingdom; ^3^Department of Molecular and Cell Biology, University of Leicester, Leicester, United Kingdom; ^4^Toxicology Department at the Centre for Radiation, Chemical and Environmental Hazards, Public Health England, Oxfordshire, United Kingdom; ^5^Department of Medicine, Campus Bio-Medico University of Rome, Rome, Italy; ^6^Department of Obstetrics and Gynaecology, Sidra Medicine, Women's Wellness and Research Center, HMC, Doha, Qatar

**Keywords:** anandamide, biomarker, endocannabinoid, endometrial cancer, prediction

## Abstract

**Objective:** To identify new biochemical markers for endometrial cancer (EC). Recent evidence suggests that members of the endocannabinoid system (*N*-acylethanolamines) that bind to and activate receptors that are dysregulated in EC are involved in this tumour's biology. These observations suggest increased *N*-acylethanolamine levels in the tissue that might appear in plasma and could be used as disease biomarkers.

**Methods:** *N*-arachidonoylethanolamine (anandamide, AEA) and the *N*-acylethanolamine substances, *N*-oleoylethanolamine (OEA), and *N*-palmitoylethanolamine (PEA) were quantified in plasma and endometrial tissue collected from 31 EC and seven atrophic controls using UHPLC-MS/MS. Receiver-operating characteristics (ROC) and logistic regression were used to determine diagnostic accuracy. Cannabinoid receptor 1 (CB1) and 2 (CB2) protein levels were determined by specific immunohistochemistry and histomorphometric analyses. Correlations between plasma and tissue levels of the three *N*-acylethanolamines and tissue levels of the three *N*-acylethanolamines and CB1 and CB2 receptor expression levels were determined using correlation analysis.

**Results:** Plasma and tissue AEA and PEA levels were significantly (*p* < 0.05) higher in EC than controls whilst OEA levels were significantly elevated in type 1 EC tissues but not in plasma. There were significant positive correlations between plasma and tissue levels of AEA (*R*^2^ = 0.302, *p* = 0.008) and PEA (*R*^2^ = 0.182, *p* = 0.047), but not for OEA (*R*^2^ = 0.022, *p* = 0.506). The diagnostic accuracies for EC were: sensitivity of 53.3%, specificity of 100% for plasma AEA (>1.36 nM); sensitivity of 73.3%, specificity of 100% for plasma PEA (>27.5 nM); and sensitivity of 93.3%, specificity of 28.6% for plasma OEA (>4.97 nM). Logistic regression increased the area under the ROC curve (AUC) from 0.781 for AEA, 0.857 for PEA, and 0.543 for OEA to a combined AUC of 0.933 for EC diagnosis. Significant inverse correlations between tissue AEA (*R*^2^ = 0.343, *p* = 0.003) and PEA (*R*^2^ = 0.384, *p* < 0.0001) levels and CB1 expression were observed. No correlation between tissue levels of OEA and CB1 and tissue levels of any of the three *N*-acylethanolamines and CB2 protein expression were observed, except in the type 1 EC patients.

**Conclusion:** Since plasma AEA and PEA are significantly elevated in patients with EC and a reflection of production by the endometrial tumour, then these lipids have the potential to be useful biomarkers for the early diagnosis of EC.

## Introduction

Despite improvements in the overall survival rates of endometrial cancer (EC), its incidence has risen by ~40% and mortality by 20% since 1999 (1–4). This disease is often diagnosed at an early stage, but ~20% of women present with advanced disease. Worldwide, 320,000 new cases were diagnosed in 2012 (5) and in the USA, it is the most common gynaecological malignancy, with over 50,000 new cases and almost 8,600 deaths reported each year (6). In the UK, it is the 4th most common cancer in women (4) and because of an ageing population and increasing obesity rates, its incidence is rising rapidly.

Current methods for EC diagnosis rely primarily on invasive techniques (biopsy) with current biochemical screening methods being unreliable (7). Post-menopausal bleeding is often the symptom whose investigation leads to diagnosis in most cases, but for a significant number of women this represents late presentation of disease. Furthermore, the early detection of EC is hampered by the lack of validated biomarkers (1, 5, 6). There is therefore a need for the identification and evaluation of novel validated biomarkers that are ideally linked to the biology of EC. Recently, a relationship between the endocannabinoid system and other forms of cancer has emerged (8–10). This system consists of a family of signalling lipids called *N*-acylethanolamines (NAE), of which the endocannabinoid *N*-arachidonoylethanolamine (anandamide; AEA) and the endocannabinoid-like compounds, *N*-oleoylethanolamine (OEA) and *N*-palmitoylethanolamine (PEA), are the best characterised (10) with respect to their receptors and the enzymes responsible for their synthesis and degradation (11, 12). Where this relationship has been investigated, it has provided an improved understanding of the biology of various tumours (13, 14). Such a relationship with ECs, however, remains relatively unexplored (8, 15). We have recently shown that the expression of cannabinoid receptors 1 and 2 (CB1 and CB2, respectively) is reduced in endometrial cancer tissue when compared to that of women of a similar age without EC (16). We therefore hypothesised that the lack of CB1 and CB2 protein in the endometrial cancer tissue might result in compensatory local increases of tissue NAE levels and that plasma concentrations of the NAEs might be a surrogate marker of tissue levels (as the NAEs are released from the tissue). Furthermore, we believe that an understanding of this relationship might eventually provide potential novel biomarkers and treatments for EC.

Here, we evaluate this relationship by quantifying levels of the three NAEs, AEA, OEA, and PEA, in the endometria of women with EC and normal controls and compare those levels to the women's plasma concentrations. We also compare the levels of these NAEs with CB1 and CB2 proteins in the same patients. From these data, we propose that plasma concentrations have the potential to be surrogates for tissue NAE levels and therefore possible good biochemical markers for EC.

## Materials and Methods

### Study Population

Volunteers were women undergoing surgical treatment (hysterectomy and bilateral salpingoophorectomy) for either endometrial cancer (EC group) or benign conditions, such as uterine prolapse (control group) at the University Hospitals of Leicester National Health Service Trust. All women gave signed written informed consent to take part in the study, which was approved and conducted according to the guidelines of the Leicestershire and Rutland Ethics Committee. The exclusion criteria included being on hormonal treatment [e.g., hormone replacement therapy or the levonorgestrel intrauterine system (Mirena^®^ Coil)], on prescription or recreational drugs (including marijuana); suffering from chronic medical conditions or any other form of cancer or a smoker. The cancer volunteers (EC group) were categorised according to the preliminary histology of the endometrial biopsies obtained at standard hysteroscopy and confirmed after surgery. All of the women in the EC group had stage 1 disease. The final histopathological categorisation into type and grade was made by the hospital Histopathology Department based on FIGO criteria. Surgery was performed within 2 weeks of the diagnosis in all cases.

### Plasma Collection

Venous blood samples were obtained from the antecubital vein into vacutainer tubes containing EDTA (Sarstedt, Leicester, UK). Plasma was separated after centrifugation at 1200 × g for 30 min at 4°C within 60 min of collection and immediately transferred into 7 mL Kimble scintillation vials (Kinesis, St. Neots, UK) and stored as 2 mL aliquots at −80°C prior to plasma lipid extraction and quantification.

### Endometrial Tissue Collection

Following hysterectomy, fresh uteri were placed on ice, transferred immediately to the Histopathology Department, where normal and tumour tissue biopsies were obtained by a senior gynaecological oncology histopathology consultant and fixed in 10% formalin for confirmation of the preliminary histological diagnosis. Additional biopsies of normal and tumour tissue were collected, washed (to remove blood) with sterile 1X phosphate buffered saline (PBS) and placed in separate sterile polypropylene tubes. These were then immediately transported to the laboratory in liquid nitrogen and stored at −80°C for later lipid extraction and NAE quantification. Power analysis of pilot data (17) indicated that to determine a 50% difference in the means of the NAE measurements with α = 0.05 and β = 0.8, a minimum of 3, 5, and 4 clinical samples for AEA, OEA, and PEA were required. Biopsies were collected until these limits were exceeded.

### Extraction and Quantification of the NAEs

NAE extraction from plasma was by a solid-phase extraction (SPE) method (18, 19) and for tissues (normal and tumour) by our modified SPE published method for solid tissues (20). For plasma (0.5 mL), deuterated AEA-d8 (2.5 pmol/mL), OEA-d2 (2.5 pmol/mL), and PEA-d4 (5 pmol/mL) internal standards (Cayman Chemical, Cambridge, UK) were added and then diluted to 1 mL with deionised water prior to loading onto an Oasis HLB cartridge (Waters Ltd, Elstree, UK). For endometrial tissues, AEA-d8 (12.5 pmol/g), OEA-d2 (12.5 pmol/g), and PEA-d4 (25 pmol/g) and 1 mL phosphoric acid (5% v/v) were added to ~100 mg of tissue. This mixture was then diluted with 1 mL deionised water, homogenised using a TissueRuptor (QIAGEN, Manchester, UK), and centrifuged at 1500 × g for 30 min at 4°C. The supernatant was transferred to a fresh tube and then loaded onto preconditioned Oasis HLB cartridge for the lipid extraction (as for plasma). After washing twice, lipids were eluted into acetonitrile, evaporated to dryness under a gentle stream of nitrogen, and re-suspended in acetonitrile (80 μL) ready for analysis. Duplicate 0.5 mL aliquots were processed and each extract analysed in triplicate. The quantification of NAEs was performed on an ultra-HPLC-tandem mass spectrometry (UHPLC-MS/MS) system, consisting of an Acquity ultra-HPLC system in line with a Quattro Premier tandem mass spectrometer (Waters Corp, Milford, Massachusetts, USA). The finer points of UHPLC-MS/MS gradient conditions and transitions used have been reported previously (18–20).

### Measurement of CB1 and CB2 Protein Levels

CB1 and CB2 protein levels were determined using immunohistochemistry on samples, identified by a gynaecology histopathologist as being representative of normal atrophic endometrium, and of type 1 and type 2 EC. Staining was performed using receptor-specific primary antibodies and subsequent histomorphometric analyses. Briefly, formalin-fixed sections of tissue were stained with CB1 (catalogue number C1108) and CB2 (catalogue number C1358) antibodies purchased from Sigma Life Science (Poole, Dorset, UK) and used within standard immunohistochemistry (IHC) protocols, with antibody amplification and 3,3'diaminobenzidine staining, as described (16). Histomorphometric analyses were performed using 10 randomised fields on each specimen with ImageScope software (Aperio UK Ltd., Axbridge, Somerset, UK) to generate an unbiased protein H-score, as described (16, 21–23). Specificity controls for the technique were as described (16). Prior power analysis for IHC studies (24) indicated that a minimum of six specimen are required for any histomorphometric analyses. Accordingly, we selected six atrophic, six each of grade 1, grade 2, and grade 3 type 1 EC and all four type 2 EC samples for these studies.

### Statistical Analysis

Statistical analysis was performed using Prism version 6:00 for Windows (Graph-Pad Software, San Diego, CA; www.graphpad.com). Demographic data are presented as mean ± SD and data not following a Gaussian distribution are expressed as median and interquartile ranges (IQR). Comparison between groups was performed using Mann-Whitney *U*-test. Correlations were determined using Spearman correlation analysis. To assess the diagnostic potential of each biomarker, receiver-operating characteristic (ROC) curves were plotted and the areas under curves (AUC) with 95% confidence intervals (95% CI), sensitivity, and specificity values calculated. Logistic regression was performed to indicate the probability of diagnosing EC using the combinations of plasma AEA, PEA, and OEA concentrations. Both ROC and logistic regression statistical analysis were performed using MedCalc version 15.5 (Ostend, Belgium). A *p*-value of <0.05 was considered statistically significant.

## Results

A total of 31 volunteers with cancer were studied: 27 had type 1 endometrioid adenocarcinoma and four had type 2 EC (two serous, one carcinosarcoma, and one clear cell carcinoma). Among those with type 1 EC, 12 had grade 1, 11 had grade 2, and four had grade 3 disease. In addition to tissue biopsies, only 15 patients with EC consented to provide plasma samples (12 with type 1 EC and 3 with type 2 EC). Seven volunteers with an atrophic endometrium were recruited as controls; all of these provided plasma samples.

### Patient Characteristics

The mean (± SD) ages of the women studied were 60.7 ± 4.3 years for the controls (*n* = 7), 65.5 ± 10.2 years for type 1 EC (*n* = 27) and 56.8 ± 7.9 for type 2 EC (*n* = 4). These were not significantly different. The mean (± SD) BMI of 26.6 ± 5.9 kg/m^2^ for the controls was significantly (*p* < 0.05) lower than 34.8 ± 7.8 kg/m^2^ for type 1 EC and 38.0 ± 5.8 kg/m^2^ for type 2 EC patients. There were no significant relationships between age or BMI with either plasma or tissue NAE measurements (data not shown).

### Plasma NAE Concentrations

Plasma AEA and PEA, but not OEA, concentrations (Table 1) were significantly higher in patients with EC than in the controls (*p* = 0.037, *p* = 0.0066, and *p* > 0.05, respectively). Plasma AEA and PEA but not OEA concentrations in those with type 1 EC were significantly higher than in the control group (*p* = 0.0129, *p* = 0.0001, *p* > 0.05, respectively). In patients with type 2 EC, NAE concentrations were similar to those of the controls.

**Table 1:** Plasma AEA, OEA, and PEA concentrations in EC and control patients.

**Ligand**	**Patient group**						
	**Control *n* = 7**	**EC (all patients) *n* = 15**	**All Type 1 EC *n* = 12**	**Type 1 EC G1 *n* = 6**	**Type 1 EC G2 *n* = 4**	**Type 1 EC G3 *n* = 2**	**All Type 3 EC *n* = 3**
AEA	1.11 (0.82–1.28)	1.52^*^ **(1.13–1.72)**	1.61^*^ **(1.23–1.77)**	1.40^ns^ (1.055–3.75)	1.67^ns^ (1.25–1.77)	1.73^ns^ (1.60–1.86)	1.04^ns^ (1.02–1.18)
OEA	6.65 (4.90–11.20)	6.78^ns^ (5.40–9.77)	7.18^ns^ (5.60–10.86)	8.54^ns^ (6.43–13.55)	6.66^ns^ (5.03–7.23)	8.22^ns^ (5.23–11.22)	5.67^ns^ (5.04–6.67)
PEA	22.76 (20.50–23.81)	41.40^**^ **(26.40–52.46)**	45.08^****^ **(32.80–96.37)**	39.08^ns^ (30.20–46.09)	112.5^***^ **(65.92–118.3)**	38.50^ns^ (30.79–46.20)	19.06^ns^ (15.45–26.42)

The data are presented as median (inter-quartile range) and plasma NAE concentrations are shown in nM. Significantly different comparisons;

* p < 0.05;

** p < 0.01;

*** p < 0.001;

**** *p < 0.0001; n.s. not significantly different when compared to the control; Mann-Whitney U-test are indicated in bold font. Numbers of samples (n) are shown. G, tumour grade*.

### Tissue NAE Levels

Tissue AEA and PEA, but not OEA, levels (Table 2) were significantly higher in cancerous than in benign tissues (*p* = 0.046, *p* = 0.032, and *p* > 0.05, respectively). Type 1 EC tissue contained significantly higher AEA, OEA, and PEA levels compared to control (benign) tissues (*p* = 0.0159, *p* = 0.0263, and *p* = 0.0015), respectively. PEA levels in type 2 EC tissues were significantly lower (*p* = 0.0061) than in control (benign) tissue, whilst the AEA and OEA levels were similar. Tissue OEA levels of all grades (1, 2, and 3) of EC were similar to those of the control endometrium, whereas those of AEA and PEA in grade 1 cancerous tissues were significantly higher (*p* = 0.0016 and *p* = 0.0002, respectively).

**Table 2:** AEA, OEA, and PEA levels in the endometrial tissues of EC and control patients.

**Ligand**	**Patient group**						
	**Control *n* = 7**	**All EC *n* = 31**	**All Type 1 EC *n* = 27**	**Type 1 EC G1 *n* = 12**	**Type 1 EC G2 *n* = 11**	**Type 1 EC G3 *n* = 4**	**All Type 2 EC *n* = 4**
AEA	1.120 (0.50–2.97)	3.929^*^ **(1.11–8.90)**	4.37^*^ **(1.39–9.18)**	9.81^**^ **(4.04–15.09)**	1.39^ns^ (0.93–6.36)	2.07^ns^ (1.06–3.70)	0.83^ns^ (0.64–1.24)
OEA	6.70 (4.08–16.16)	15.39 ^ns^ (6.41–21.20)	16.71^*^ **(8.77–23.88)**	19.11 ^ns^ (13.82–26.83)	16.71 ^ns^ (6.85–45.00)	9.00 ^ns^ (4.80–16.28)	6.311 ^ns^ (6.17–10.31)
PEA	43.20 (33.91–65.49)	115.9^*^ **(53.06–266.3)**	172.1^**^ **(86.73–278.2)**	272.2^****^ **(184.5–380.0)**	95.36 ^ns^ (53.06**–**172.1)	92.35 ^ns^ (36.89**–**171.0)	19.12^**^ **(14.88–22.26)**

The data are presented as median (inter-quartile range). The values of NAE levels are shown as pmol/g of tissue. Significantly different comparisons;

* p < 0.05;

** p < 0.01;

*** p < 0.001;

**** *p < 0.0001; n.s. not significantly different when compared to the control; Mann-Whitney U-test are indicated in bold font. Numbers of samples (n) are shown. G, tumour grade*.

### Correlations Between Plasma Concentrations and Endometrial Tissue Levels of AEA, OEA, and PEA

**Figure 1:**
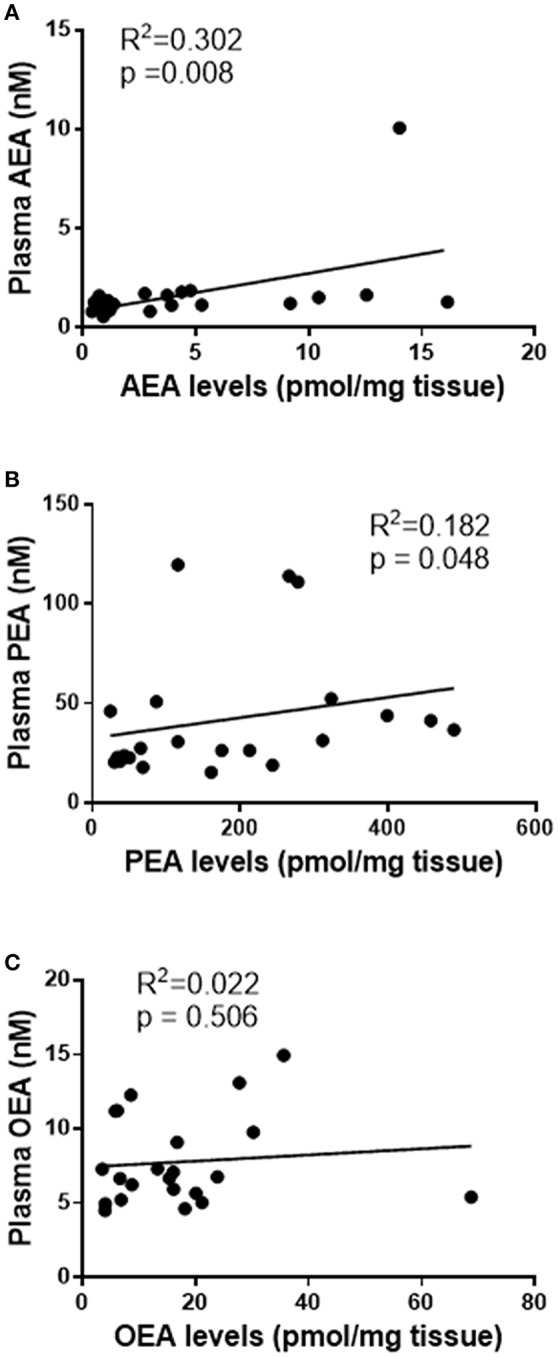
Correlation curves showing the relationships between tissue levels of AEA, PEA, and OEA with plasma concentrations. The relationships between plasma concentrations and tissue levels (*n* = 22) of AEA **(A)**, PEA **(B)**, and OEA **(C)** are shown. The indicated *R*^2^ and *p*-values were obtained using Spearman correlation analysis.

Having determined the levels of NAEs in plasma and tissues, the next logical analysis was to determine if these two parameters were related. Stratification into the three patient groups indicated no significant group-specific correlations between any of the tissue or plasma NAE measurements. However, when combined into a single study cohort, there was a statistically significant correlation between plasma and tissue AEA levels [*R*^2^ = 0.302, *p* = 0.008; *n* = 22, (Figure 1A)] and plasma and tissue PEA levels (*R*^2^ = 0.182, *p* = 0.048, *n* = 22) (Figure 1B). There was, however, no statistically significant correlation between plasma and tissue OEA levels (*R*^2^ = 0.022, *p* = 0.506, *n* = 22) (Figure 1C).

### Correlations Between Endometrial Tissue Levels of AEA, OEA, PEA, and CB1 or CB2 Levels

**Figure 2:**
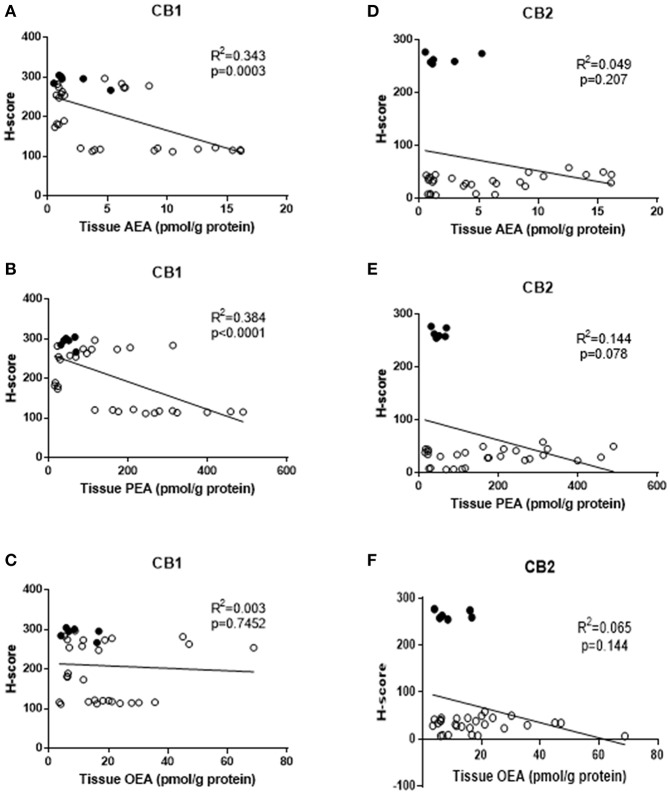
Correlation between tissue levels of AEA, PEA, and OEA with tissue protein expression levels. The relationships between tissue levels of AEA and CB1 or CB2 protein **(A,D)**, tissue levels of PEA and CB1 and CB2 protein **(B,E)** and OEA and CB1 or CB2 protein **(C,F)** are shown. The data for atrophic controls (*n* = 6) are shown by filled circles and those for the EC patients (*n* = 28; 24 Type 1 and 4 type 2 EC) by open circles. The indicated *R*^2^ and *p*-values were obtained using Pearson correlation analysis.

We hypothesised that reduced expression of cannabinoid receptor proteins (CB1 and/or CB2) might be related to the observed increases in tissue AEA, OEA, and PEA levels. A simple correlation analysis of H-score measurements of CB1 immunoreactivity indicated a significant inverse correlation between tissue AEA levels and CB1 protein levels [*R*^2^ = 0.343, *p* = 0.0003, *n* = 34 (Figure 2A)] and between tissue PEA levels and CB2 protein levels [*R*^2^ = 0.384, *p* < 0.0001, *n* = 34, (Figure 2B)]. There was, however, no statistically significant correlation between tissue OEA levels and CB1 protein levels (*R*^2^ = 0.003, *p* = 0.745, *n* = 34) (Figure 2C). Additionally, there were no significant correlations between any of the tissue levels of AEA, PEA or OEA, and CB2 protein levels (Figures 2D–F, respectively). Stratification of these data into the three patient groups indicated significant inverse correlations only between CB1 expression and tissue AEA levels in the type 1 EC patients (*r* = −0.607, *p* = 0.007); CB1 expression and tissue PEA levels in type 1 EC patients (*r* = −0.719, *p* < 0.001) and a positive correlation between CB1 expression and PEA levels in type 2 EC patients (*r* = 0.676, *p* = 0.031). By contrast, only significant correlations between CB2 expression and tissue AEA (*r* = 0.746, *p* < 0.001) and PEA levels (*r* = 0.600, *p* = 0.008) were demonstrated in type 1 EC patients. All other combinations showed no significant associations.

### Plasma Concentration ROC Curves

**Figure 3:**
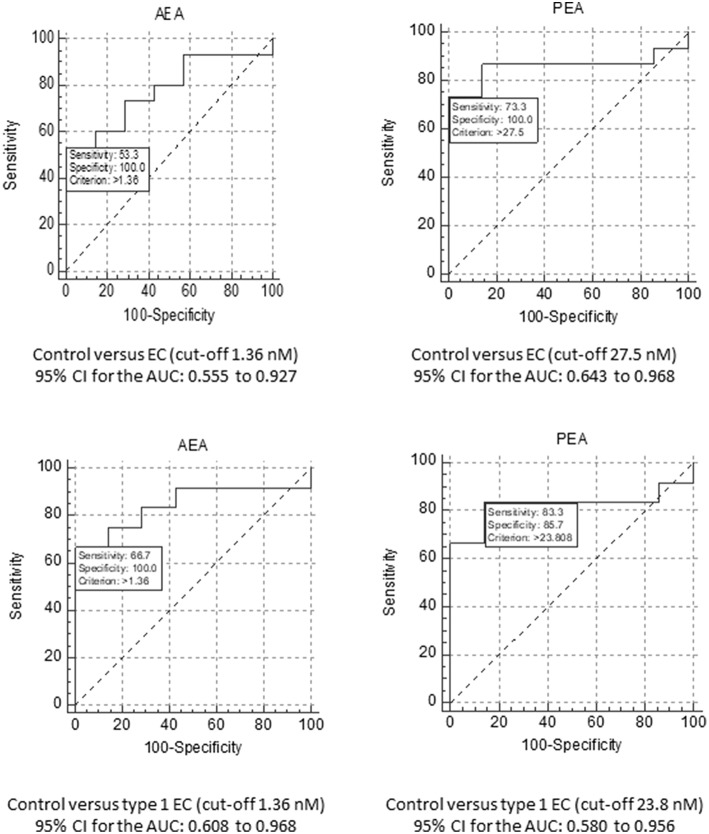
ROC analyses for AEA and PEA. The upper panels show the ROC analyses for AEA **(Left)** and PEA **(Right)** for the diagnosis of EC, using a cut-off plasma concentration of 1.36 nM for AEA and 27.5 nM for PEA. The data also show the sensitivity and specificity values for these biomarkers together with the value for the area under the ROC curve and their 95% confidence intervals (CI). The lower panels show similar analyses, but for the comparison of EC grade 1 tumour patients only compared to the controls. In this case, the cut-off plasma concentration remained at 1.36 nM for AEA and changed to 23.8 nM for PEA.

ROC curves were constructed for plasma NAEs from 22 (15 EC plus 7 control) patients (Figure 3). Plasma concentrations of AEA had the best diagnostic accuracy for all types of EC with an AUC of 0.781. At a cut-off value of 1.36 nM, the sensitivity was 53.3% and specificity was 100% for the diagnosis of EC (*p* = 0.0734) for a likelihood ratio of 5.25. When the ROC analysis was applied to the diagnosis of type 1 EC only (*n* = 19; 12 EC and seven controls) the AUC increased to 0.845 for a sensitivity of 66.7% and a specificity of 100% (*p* = 0.0246; likelihood ratio 4.50).

The ROC for plasma PEA concentration (*n* = 22; 15 EC and seven controls) revealed the best diagnostic accuracy for all types of EC with an AUC of 0.857 (Figure 3). For a cut-off of 27.5 nM, its sensitivity was 73.3% and the specificity was 100% (*p* < 0.0001; likelihood ratio 6.07). Focussing only on type 1 EC (*n* = 19; 12 EC and seven controls), the plasma PEA cut-off value became 23.8 nM for the seven controls and 12 type 1 EC plasma PEA samples. The AUC was 0.821 for the diagnosis of type 1 EC with a sensitivity of 83.3% and a specificity of 85.7% (*p* = 0.0026; likelihood ratio 5.83).

When plasma OEA concentration was modelled for diagnostic accuracy for all types of EC, an AUC of 0.543 was generated, for a cut-off value of 4.965 nM, for a sensitivity of 93.3% and a specificity of 28.6% (*p* > 0.05), with a likelihood ratio of 1.307. When the modelling was confined to the diagnosis of type 1 EC only (*n* = 19), the AUC increased to 0.583 with an optimal cut-off value of 6.655 nM, providing a sensitivity of 66.7% and a specificity of 57.1% (*p* = 0.0026; likelihood ratio of 1.56).

When the plasma concentrations of AEA and PEA were combined to generate ROC curves for the diagnosis of all types of EC, the AUC increased from 0.781 for AEA and 0.857 for PEA to 0.924. For type 1 EC alone, the AUC increased to 0.917. When all the three NAEs (AEA, PEA, and OEA) were combined, the AUC of the ROC curve for the diagnostic accuracy of all types of EC increased from 0.781 for AEA, 0.857 for plasma PEA and 0.543 for plasma OEA to 0.933. When the analysis was repeated but only for type 1 EC, a value similar to that obtained with plasma concentrations of AEA and PEA combined but with OEA omitted (0.917) was generated.

## Discussion

The levels of AEA and PEA were significantly higher in the plasma and tissues of patients with EC when compared to age-matched controls. The levels of these two NAEs were also significantly inversely correlated with CB1 protein levels that decrease in the endometrial cancer tissue, suggesting that loss of this particular receptor results in increased availability of AEA and PEA in the tissue and subsequently in plasma. These findings suggest that these NAEs may be involved in the pathogenesis of EC. These data are similar to those produced by Guida et al. (25), in that both tissue AEA and PEA levels were elevated in EC, but differed in their absolute measurements, presumably because of differences in tissue selection (Guida et al. describe contamination of tumour with normal tissue as being an issue in their study). Guida et al., also reported loss of CB1 receptor protein, but did not relate the tissue AEA and PEA levels to changes in receptor expression. Previous studies have demonstrated that the levels of the endocannabinoid 2-AG were significantly increased in endometrial (25) and other cancers (14, 26), suggesting that other endocannabinoids might be elevated in EC. An important observation in the present study was the positive correlation between plasma concentrations and tissue levels of AEA and PEA, something that, to our knowledge, has not been reported previously, even in the 2-AG study (25). This significant positive correlation between plasma AEA and PEA concentrations with tissue levels, but not that of OEA, led us to hypothesise that plasma levels of the former are surrogates of tissue levels and that these could be used as markers for diagnosis. An alternative hypothesis that the NAEs are produced in other parts of the body and accumulate in the rapidly metabolising tumour tissue is not supported because (1) there should be a strong correlation between the plasma concentrations of all three NAEs and tissue NAEs if this was the only determinant (and there was not a strong correlation for OEA) and (2) as blood volume increases then tissue concentration should also increase (this was not observed in our cohort). Examination of correlates between BMI (a measurement of patient size and blood volume), which is known to increase EC risk in our patients (27) and tissue NAE concentrations, indicated there was no relationship between these parameters in any of the patient groups. Since the endometrium expresses the enzymes that regulate the tissue and plasma concentrations of these (28) and other endocannabinoids (25), then it is highly likely that plasma NAE concentrations are a reflection of the balance between direct NAE synthesis and degradation in the endometrium.

The ROC analyses in the present study were designed to identify the possible cut-off values for the plasma NAE concentrations and thus provide support to the notion that these plasma biomarkers could have the potential to be used as clinical biomarkers in the future. We have thus performed the “detection” stage of ROC analysis for the NAEs as plasma biomarkers; the “validation” stage will need to be performed in a double blind randomised clinical trial based on the plasma cut-off values and predictability values presented herein. The specificity and sensitivity of plasma AEA and PEA concentrations in discriminating between patients with benign endometria and those with EC appear to be superior to other serum biomarkers that have been published, such as encapsulated miRNA species, cyclophilin A and HE-4 (29), as evidenced by logistic regression analysis. The latter shows that the combination of these ligands (AEA and PEA) gives a better detection (AUC of 0.933) than either NAE alone. Previously reported serum markers of EC, such as CA125 and CA15-3 only produced 11–34 and 47% specificity values for advanced stage tumours, respectively (7), suggesting that other markers or combinations would be useful. Indeed, the addition of HE-4 to the analysis of CA-125 (30) produced a 12.9% increase in accuracy for the diagnosis of early (type 1) EC, and L1CAM when added to mutant p53 increased the probability of detecting more advanced EC (31). This background prompted us to combine AEA, PEA, and OEA data. The fact that there was no significant change in plasma OEA concentrations or correlation with tissue levels, suggests that this NAE is not useful as a biomarker either alone or in combination with AEA and PEA, but may point towards a possible mechanism in EC pathogenesis. The “entourage effect” is a well-known, but poorly characterised phenomenon in endocannabinoid biology (32), whereby the NAE degradative enzyme fatty acid amide hydrolase (FAAH) preferentially degrades one of the substrates, whilst sparing one or more others (33, 34). In the malignant tissue of the endometrium, it appears that OEA is the preferential target of endometrial FAAH, which might explain the lack of correlation between plasma concentrations and tissue OEA levels (Figure 1), and the lack of difference between plasma concentrations and tissue levels in the EC patients (Tables 1, 2). This hypothesis will need to be tested further, but is highly likely, based on current observations, that the levels and function of FAAH will also be altered in EC, as they are in other tumours (35, 36).

An alternative hypothesis relates to dysregulation of the endocannabinoid system by sex steroid hormones. The observed higher AEA and PEA levels in the tissue (and thus in the plasma) might be due to an imbalance in the estrogen to progesterone ratio, a known factor involved in the development of EC. This was elegantly demonstrated in a mouse study (37), where endocannabinoid synthesis was found to be directly regulated by estradiol. Furthermore, AEA plasma concentrations have been demonstrated to directly correlate with human endocannabinoid biosynthesis in response to estrogen, but not progesterone (38). In addition, estrogen reduces (39) and progesterone increases (40) human FAAH activity and expression, which correlates with observed AEA and PEA tissue levels and the endometrioid nature of the type 1 EC highlighted in the present study (41, 42). The outcomes of these changes are difficult to predict *in vivo*, but increased mitotic activity and enhanced DNA replication in endometrial cells caused by an aberrant estrogen to progesterone ratio (43) may also affect the expression of cannabinoid receptors, as has been reported by Guida et al. (25). A role for estrogens in the pathogenesis of type 1 EC through the production of NAEs is supported by the observation that AEA and PEA were not elevated in type 2 EC, which is known to be less responsive to endogenous and exogenous estrogens (44). The reduction of PEA in the endometrium of patients with type 2 EC, suggest different pathogenic pathways involved in type 2 EC that are distinct to that of type 1 EC where PEA levels increase. Whether this is a cause or effect is something that needs further investigations.

NAEs are increasingly being recognised to play multiple roles in the regulation of key processes involved in the development of cancer. For example, they are reported to induce apoptosis (45, 46), cell cycle arrest (47, 48), and the inhibition of angiogenesis and metastasis (49). Furthermore, endocannabinoid signalling in malignant cells may differ to that of their “normal” counterpart (50). These data suggest that NAEs are not only potentially useful for the diagnosis of EC, but that factors that control the synthesis and degradation of these lipids, or their activation of receptor expression, could be key to an effective non-invasive treatment, as has been proposed for other cancers (8, 50).

Although the number of patients recruited to the present study may be considered a limitation, our number of those with EC type 1 is similar to that of Guida et al. (25) hence our results are thus supportive of AEA and PEA being intimately involved in the pathogenesis of EC type 1. The number of EC type 2 patients was smaller than for EC type 1, which is a reflection on the prevalence of the two types of EC in the UK population (44), but such data could provide the basis for further research, which would be important so as to substantiate the present findings. For example, the significance of the data presented here suggest that plasma NAEs and of other endocannabinoids (8, 15) will be useful in the diagnosis and possible management of EC patients, and may have both pathophysiological and diagnostic consequences for EC.

## Ethics Statement

This study was performed within National Health Service Research Governance Framework guidelines. All subjects gave written informed consent in accordance with the Declaration of Helsinki. The protocol was approved by the Leicestershire, Northamptonshire and Rutland Research Ethics Committee (LREC Reference Number: 06/Q2501/49).

## Author Contributions

JK designed the study, while TA recruited the volunteers, collected the tissues, and undertook the quantification with supervision from TM. TA and AT performed the data analysis with input from JK and TM. TA produced the initial draft of the manuscript and AT, TM, MM, and JK all contributed to its revisions. JK is the senior supervisor and guarantor of the study.

### Conflict of Interest Statement

The authors declare that the research was conducted in the absence of any commercial or financial relationships that could be construed as a potential conflict of interest.
